# Absorption-Enhanced Ultra-Thin Solar Cells Based on Horizontally Aligned p–i–n Nanowire Arrays

**DOI:** 10.3390/nano10061111

**Published:** 2020-06-04

**Authors:** Xueguang Yuan, Xiaoyu Chen, Xin Yan, Wei Wei, Yangan Zhang, Xia Zhang

**Affiliations:** 1 State Key Laboratory of Information Photonics and Optical Communications, Beijing University of Posts and Telecommunications, Beijing 100876, China; yuanxg@bupt.edu.cn (X.Y.); Cxy.1@bupt.edu.cn (X.C.); zhang@bupt.edu.cn (Y.Z.); xzhang@bupt.edu.cn (X.Z.); 2 School of Mechanical and Electric Engineering, Guangzhou University, Guangzhou 510006, China; wei@gzhu.edu.cn; 3 Photonics Research Centre, Department of Electronic and Information Engineering, The Hong Kong Polytechnic University, Hung Hom, Kowloon, Hong Kong, China

**Keywords:** horizontal nanowire array, absorption-enhanced, solar cell, refractive index difference, GaAs

## Abstract

A horizontally aligned GaAs p–i–n nanowire array solar cell is proposed and studied via coupled three-dimensional optoelectronic simulations. Benefiting from light-concentrating and light-trapping properties, the horizontal nanowire array yields a remarkable efficiency of 10.8% with a radius of 90 nm and a period of 5 radius, more than twice that of its thin-film counterpart with the same thickness. To further enhance the absorption, the nanowire array is placed on a low-refractive-index MgF_2_ substrate and capsulated in SiO_2_, which enables multiple reflection and reabsorption of light due to the refractive index difference between air/SiO_2_ and SiO_2_/MgF_2_. The absorption-enhancement structure increases the absorption over a broad wavelength range, resulting in a maximum conversion efficiency of 18%, 3.7 times higher than that of the thin-film counterpart, which is 3 times larger in GaAs material volume. This work may pave the way for the development of ultra-thin high-efficiency solar cells with very low material cost.

## 1. Introduction

Semiconductor nanowires (NWs) have shown great potential in low-cost high-performance solar cells [1,2,3,4]. The unique quasi-one-dimensional NW enables the realization of solar cells with different structures, including axial, radial, or hybrid p–i–n or Schottky junctions aligned vertically or horizontally [5,6,7,8]. The vertically aligned NW solar cell has been widely reported and a remarkable efficiency of 15.3% has been obtained in an axial p–i–n GaAs NW array solar cell [5]. The horizontal NW solar cell is achieved by transferring the NWs from the substrate onto another rigid or flexible supporter, in which the long axis of the NW is parallel with the supporter, and the incident light enters the NW through the side surface. Although the light-concentrating properties of the horizontal NW are weaker than those of the vertically aligned one, the horizontal NW still possesses substantial advantages over the thin-film counterpart [9]. Particularly, the horizontal NWs have a much thinner thickness in comparison with the vertically aligned NWs and thin films due to the ultra-small cross-section of NWs, enabling the realization of ultra-thin solar cells. Moreover, the horizontal NWs are easily integrated on the glass and plastic substrates for transparent and flexible photovoltaics, demonstrating a promising perspective for next-generation smart solar energy harvesting devices [10]. Up to date, horizontal NW solar cells based on various materials, including Si, GaAs, InGaP, CdS, and InP, have been demonstrated [11,12,13,14,15,16,17,18]. Among those materials, GaAs is particularly promising for photovoltaic applications as its band gap matches the solar spectrum well. Colombo et al. have reported a single horizontal p–i–n radial GaAs NW solar cell with an efficiency of 4.5% [13]. Han et al. have fabricated a single horizontal GaAs NW Schottky barrier solar cell with an efficiency of 2.8% [14]. Although those devices have shown advantages in dimension and flexibility, the conversion efficiency is still very low and requires further improvement.

In comparison with vertically aligned NWs, the light-trapping and light-concentrating effects in horizontally aligned NWs are weaker due to the much smaller thickness and lower filling ratio, leading to relatively strong reflection and low absorption of incident light [19]. Hence, the structural parameters of horizontal NWs, such as the diameter and period, should be precisely designed for optimal optical absorption. For example, too small a period leads to a strong competition of optical absorption for adjacent NWs, while too large a period results in a waste of the incident light. Moreover, to overcome the insufficient absorption of the ultra-thin NW array, the underlying substrate as well as the surroundings should be engineered to achieve multiple re-absorption. However, compared with the widely studied vertically aligned NW arrays, detailed study and optimization of horizontally aligned NW array (HNA) solar cells are extremely limited [20,21,22].

In this paper, an HNA solar cell is proposed and optimized in detail. The device consists of horizontally aligned NWs with axial p–i–n junctions placed on a dielectric substrate. The optical absorption properties and photovoltaic performance of devices are investigated via the finite-difference time-domain (FDTD) and finite element method (FEM), respectively. The size and period of NWs are tuned to obtain optimal light absorption. With a radius of 90 nm and period of 5 radius, the HNA solar cell yields a remarkable efficiency of 10.8%, more than twice that of its thin-film counterpart with the same thickness. To achieve multiple reabsorption of light, the horizontal NWs are placed on a low-refractive-index MgF_2_ substrate and capsulated in SiO_2_. Due to the index difference between air/SiO_2_ and SiO_2_/MgF_2_, the light striking the substrate is reflected at the SiO_2_/MgF_2_ and SiO_2_/air interfaces and reabsorbed by the NWs. A maximum conversion efficiency of 18% is obtained for NWs capsulated in a 350-nm-thick SiO_2_ layer, enabling the realization of high-efficiency solar cells with ultra-small thickness and ultra-low material cost.

## 2. Methods

**Figure 1:**
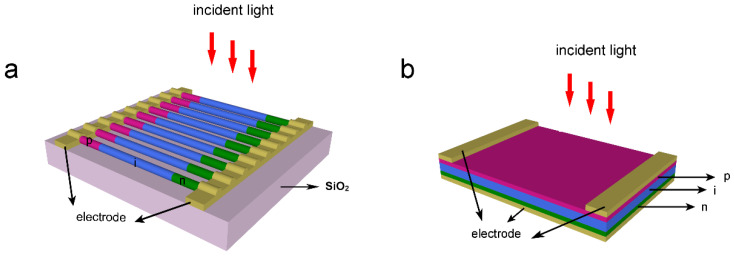
Schematic diagram of (**a**) the horizontally aligned nanowire array (HNA) structure and (**b**) the thin-film counterpart.

Figure 1a is the model of the HNA solar cell, which consists of multiple horizontal NWs arranged parallel to each other. Each NW consists of a 4-μm-long GaAs NWs lying on a SiO_2_ substrate with a thickness of 600 nm. Each NW contains an axial p–i–n junction, in which the p- and n-regions have the same length of 1 μm and are uniformly doped to 3 × 10^18^ and 1 × 10^17^ cm^−3^, respectively. All the NWs are connected in parallel by electrodes placed on the p- and n-sections of each NW. A practical HNA solar cell can be fabricated as follows. Axial p–i–n GaAs NWs are grown by molecular beam epitaxy or metal organic chemical vapor deposition, with Zn and Si as the p- and n-dopant, respectively. As-grown NWs are transferred onto a 600-nm-thick SiO_2_ layer by sonication with ethanol as the carrier. The horizontal NWs are then aligned in parallel by micromanipulations. Finally, metal electrodes are defined on both ends of each NW by photolithography and electromagnetic sputtering followed by a “lift-off” process. The light illuminates the NWs from the top. Figure 1b shows the thin-film counterpart with a planar p–i–n structure, which has the same thickness as the HNA. In the simulations, the diameter and the period of NWs are changed to achieve optimum photoelectric conversion performance.

The performance of the proposed structures is simulated by Sentaurus TCAD. Optical properties of the structure are investigated through the Sentaurus Electromagnetic Wave (EMW) Solver module package. The minimum cell size of the FDTD mesh is set to 5 nm, and the number of nodes per wavelength is 20 in all directions. By placing periodic boundary conditions, the simulations can be carried out in a single unit cell to model the periodic array structure. In order to save the resources and time required for the calculation, the substrate thickness is limited to 0.6 μm. However, by using a perfect match layer (PML) adjacent to the substrate, the transmission light is totally absorbed, which enables us to model a semi-infinite substrate. The wavelength-dependent complex refractive indexes of GaAs, SiO_2_, and MgF_2_ are obtained from References [23,24,25]. We use a plane wave defined with power intensity and wavelength values from a discretized AM 1.5 G solar spectrum to model the sunlight. The transverse electric (TE) and transverse magnetic (TM) mode contributions are superimposed to model the corresponding unpolarized feature of sunlight. The total optical generation under the AM 1.5 G illumination can be modeled by superimposing the power-weighted single-wavelength optical generation rates. The optical generation rate $G_{ph}$ is obtained from the Poynting vector S:

(1) $$G_{ph}=\frac{\left|\vec{\nabla }\cdot \vec{S}\right|}{2\hbar \omega }=\frac{\varepsilon^{\prime \prime }{\left|\vec{E}\right|}^{2}}{2\hbar }$$

where ħ is the reduced Planck’s constant, ω is the angular frequency of the incident light, E is the electric field intensity at each grid point, and $\varepsilon^{\prime \prime }$ is the imaginary part of the permittivity. The reflection monitor is located above the top surface of the NW array, and the transmission monitor is located at the bottom surface of the substrate to calculate the light absorbed. The amount of power transmitted through the power monitors is normalized to the source power at each wavelength. The reflectance R(λ) and transmission T(λ) are calculated by the equation:

(2) $$R(\lambda ),T(\lambda )=0.5\int real\{p{\left(\lambda \right)}_{monitor}\}dS/P_{in}(\lambda )$$

where P(λ) is the Poynting vector, dS is the surface normal, and $P_{in}(\lambda )$ is the incident source power at each wavelength. The absorption spectrum A(λ) of the whole structure is given by the following equation:

(3) A(λ)=1−R(λ)−T(λ)

For an NW array with an incomplete filling factor, the absorption efficiency is defined as the ratio of the absorption cross-section to the total NW projected area. For the electrical modeling, the 3D optical generation profiles are incorporated into the finite-element mesh of the NWs in the electrical tool, which solves the carrier continuity equations coupled with Poisson’s equation self-consistently in 3D. The doping-dependent mobility, band-gap narrowing, as well as the radiative, Auger, and Shockley–Read–Hall (SRH) recombinations are taken into consideration in the device electrical simulations. The recombination data used in the simulations are obtained from the Levinshtein model [23], which is shown in Table 1. The Arora model is adopted in the calculation of the doping-dependent mobility [26], which reads

(4) $$\mu_{dop}=\mu_{\min}+\frac{\mu_{d}}{1+{\left(N/N_{0}\right)}^{A}}$$

where *A* is 0.6273 (0.8057) and *N*_0_ is 7.345 × 10^16^ (5.136 × 10^17^) for the electrons (holes).

## 3. Results and Discussion

**Figure 2:**
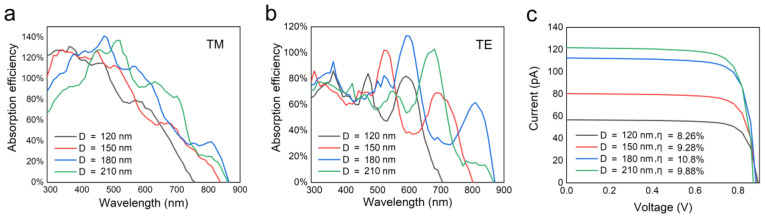
(**a**,**b**) The absorptance of the single horizontal nanowires (NWs) with different diameters for transverse-magnetic (TM) and transverse-electric (TE) polarized light, respectively. (**c**) The current–voltage characteristics of single horizontal NWs with different diameters.

The diameter-dependent absorption characteristics of a single horizontal p–i–n GaAs NW are firstly studied to find the optimal dimension of NWs used in the horizontal array. As the horizontal NW is not isotropic in the x–y plane, the absorption spectra for the TM and TE polarized light are different, as shown in Figure 2a,b. Generally speaking, TM polarization has higher absorption than TE polarization due to the enhanced optical antenna effect for each NW in TM polarization [19,27]. At some wavelengths, the absorption efficiency exceeds 100%, which is attributed to the light-concentrating effect, that is, the absorption cross-section of the NW is larger than its physical size [9]. This effect enables a high-absorption efficiency of NWs with a small dimension. As the diameter increases, the absorption peak of the NW exhibits a redshift, which is more pronounced for the TE polarized light. A similar phenomenon has also been reported in other works, which is attributed to the resonant modes in the wire that lead to strong absorption at a specific wavelength [28]. For NWs with diameters of 120 and 150 nm, the absorption cutoff wavelength is much shorter than the band-gap wavelength due to the weak lateral confinement of the long wavelength waveguide modes by the ultra-thin NWs. As the diameter increases, the absorption increases at long wavelengths but drops at short wavelengths. Hence, an NW with a moderate diameter should be more suitable to match the solar spectra and yield a high efficiency. Figure 2c shows the *I*–*V* characteristics and corresponding conversion efficiency of the single horizontal NWs with different diameters. It can be seen that as the diameter increases, the efficiency first increases and then decreases, leading to an optimal value of 10.8% at a diameter of 180 nm, which is in agreement with the absorption analysis.

**Figure 3:**
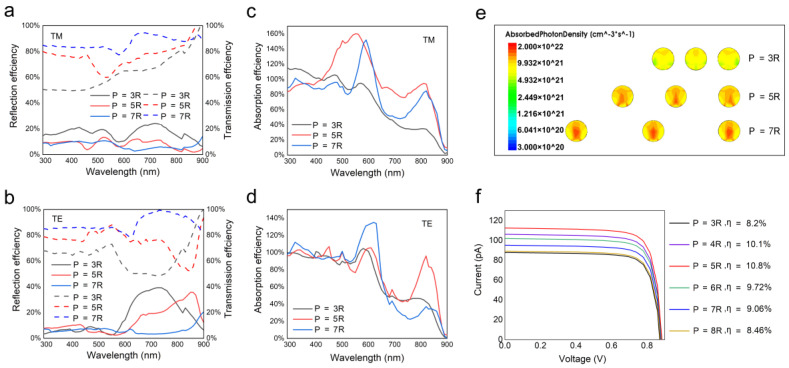
(**a**,**b**) The reflectance and transmittance of the HNA structures with different periods for TM and TE polarized light, respectively. (**c**,**d**) The absorptance of HNA structures with different periods for TM and TE polarized light, respectively. (**e**) The cross-sectional optical generation profiles of HNA structures with different periods. (**f**) The current–voltage characteristics of HNA structures with different periods.

Although the efficiency of a single NW solar cell is high, the output current is relatively low. For the purpose of practical applications, an NW array solar cell is constructed by connecting NWs in parallel. Apparently, the spacing between NWs, or in other words, the period, plays a critical role in light absorption. Here, the period is defined as the distance between centers of two neighboring NWs, indicated as the multiple of radius *p* = xR. Figure 3a,b shows the reflectance and transmittance spectra of the NW array with periods of 3, 5, and 7R for the TM and TE polarized light, respectively. It can be seen that when the period increases from 3 to 5R, the reflection decreases over nearly the whole wavelength range due to the enhanced light-trapping effect. As the period further increases to 7R, reflection is enhanced at some wavelengths, which is attributed to the increased mirror reflection by the void space. As the period increases from 3 to 7R, the transmission decreases particularly at short wavelengths due to the increased void area between NWs.

Figure 3c,d shows the absorption efficiency of the NW array with different periods for the TM and TE polarized light, respectively. It can be seen that at some wavelengths, the absorption efficiency exceeds 100%, suggesting that the light-concentrating effect also exists in the HNA structure. When the period increases from 3 to 5R, the absorption of TM polarized light is significantly enhanced over a broad wavelength range from 400 nm to the band gap, and the absorption of TE polarized light is also enhanced at long wavelengths after 750 nm. For a larger period of 7R, the absorption at long wavelengths drops for both TM and TE polarized light. Moreover, as the period increases, the absorption peak exhibits a redshift, which could be attributed to the penetration of leaky NW cavity modes into the space between NWs [29]. In order to show the absorption characteristics more directly, the cross-sectional optical generation profiles are shown in Figure 3e. For a dense array with a period of 3R, the absorption inside the NW is very weak, which is attributed to the competition of incident light by neighboring NWs and the suppression of the light-concentrating effect. This is supported by the absorption spectra shown in Figure 3c,d that show the absorption efficiency at a spacing of 3R is lower than 100% at most wavelengths. As the period is increased to 5R, the absorption is significantly enhanced over nearly the whole cross-section, suggesting that the competition of light by neighboring NWs is significantly suppressed and that each NW can collect sufficient photons. When the period further increases to 7R, the absorption is still strong at the center of cross-section but drops at the edges, which may be attributed to a weak reabsorption of light reflected at the substrate surface or scattered by neighboring NWs with such a large spacing. Figure 3f shows the current–voltage characteristics and corresponding conversion efficiency of HNA structures with different periods. In agreement with the absorption analysis, the efficiency of HNA structures first increases and then decreases as the period increases from 3 to 8R. At a moderate period of 5R, the HNA structure generates an optimum efficiency of 10.8% by considering the projected area of the NWs under AM 1.5 G illumination. 

**Figure 4:**
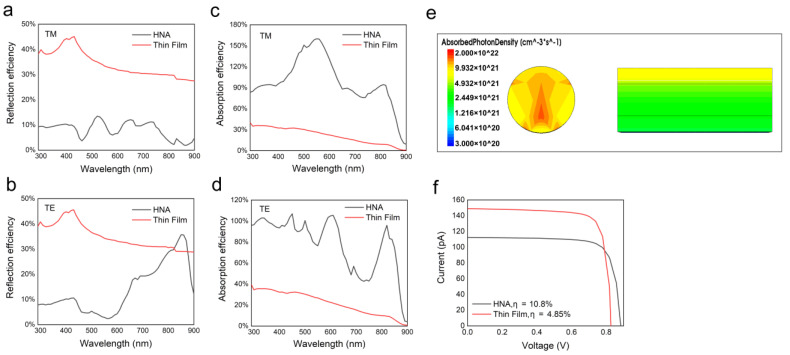
(**a**–**d**) The reflectance and absorptance of the HNA and thin-film structures for TM and TE polarized light, respectively. (**e**) Comparison of the cross-sectional optical generation profiles of the HNA and thin-film structures. (**f**) The current–voltage characteristics of the HNA and thin-film structures.

To show the advantage of the HNA structure in photovoltaics, the performance is compared with that of the thin-film counterpart. Figure 4a,b shows the reflectance of the HNA structure with an optimal period of 5R and a thin-film counterpart with the same thickness. Due to the strong mirror reflection, the reflectance of the thin film is several times higher than that of the HNA structure over the whole wavelength range. Figure 4c,d shows the absorptance of the two structures. It can be seen that the absorptance of the HNA structure is much higher than that of the thin-film counterpart, suggesting excellent light-trapping and light-concentrating properties. Figure 4e shows the cross-sectional optical generation profiles in two structures. For the thin-film structure, absorption is mainly concentrated in the top layer. Before absorbed by the active region (i-layer), the photons should pass through the top p-layer first and be absorbed. Due to the lack of an electric field in the top p-layer, the photocarriers cannot be separated effectively, and they quickly recombine before arriving at the electrodes. On the other hand, as the recombination rate strongly depends on the number of defects, the recombination of carriers in the top p-layer should be stronger due to the high doping level. In our simulations, due to a lack of doping-dependent recombination values, this effect is not considered at present. However, in practice this effect should not be ignored as it will degrade the performance of the thin-film solar cell. Based on the two factors mentioned above, photocarriers generated in the top p-layer are quickly recombined and contribute little to the photocurrent, leading to a great loss of incident light. For the axial p–i–n NW, though, the light can be directly absorbed by the active region without passing through the doped region, which is expected to enhance the absorption of the active region and raise the efficiency. Figure 4f shows the current–voltage characteristics and corresponding conversion efficiency of the HNA and thin-film structures. The conversion efficiency is calculated by the equation:

(5) $$\eta =\frac{V_{oc}\cdot I_{sc}\cdot FF}{W_{AM1.5G}}=\frac{V_{oc}\cdot I_{sc}\cdot FF}{w\cdot S_{a}}$$

where *V_oc_* is the open-circuit voltage, *I_sc_* is the short-circuit current, *FF* is the fill factor, *w* is the density of solar power, which is 0.1 W/cm^2^, and *S_a_* is the active area (projected area) of the NW, that is, the product of the diameter and the length of NW (0.18 μm × 4 μm = 0.72 μm^2^). The HNA structure yields a *V_oc_* of 0.88 V, an *I_sc_* of 113 pA, and an *FF* of 78%, resulting in a conversion efficiency of 10.8%, more than twice that of its thin-film counterpart with the same thickness.

**Figure 5:**
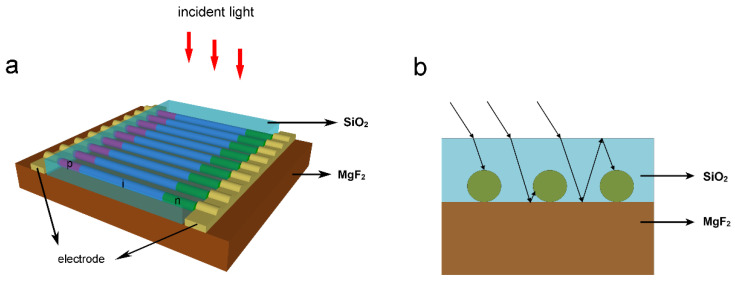
(**a**,**b**) Schematic diagram and working principle of the absorption-enhanced (AE-HNA) structure.

Although the HNA structure exhibits a higher performance than its thin-film counterpart, the efficiency is still low due to the insufficient absorption of light by the ultra-thin NWs. To further improve the performance, an absorption-enhanced (AE-HNA) structure is proposed by engineering the materials surrounding the NWs, as shown in Figure 5a. The supporting substrate is replaced by a low-refractive-index MgF_2_ film, and the NWs are capsulated in a SiO_2_ layer. It should be noted that the electrodes should be exposed to the air in order to output the photocurrent. To achieve this structure, after transferring the NWs onto the MgF_2_ substrate and depositing electrodes, a SiO_2_ layer is deposited on the NWs. The SiO_2_ at two ends is then etched to expose the electrodes. Figure 5b shows the schematic of the light transmission path. The refractive indexes of air, SiO_2_, and MgF_2_ follow. One part of incident light is directly absorbed by the NWs, while some light striking the SiO_2_/MgF_2_ interface is reflected due to the refractive index difference between SiO_2_/MgF_2_. One part of the reflected light is reabsorbed by the NWs, while other light striking the SiO_2_/air interface is reflected back to the NWs or SiO_2_/MgF_2_ interface. As a result, the absorption of NWs is significantly enhanced due to the multiple absorption paths. 

**Figure 6:**
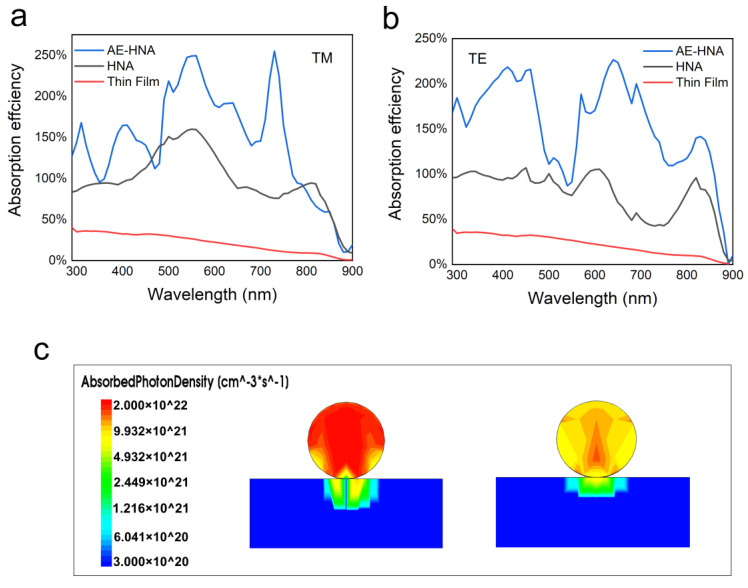
(**a**,**b**) The absorptance of the AE-HNA, HNA, and thin-film structures for TM and TE polarized light, respectively. (**c**) Comparison of the cross-sectional optical generation profiles of the AE-HNA and HNA structures.

Figure 6a,b shows the TM and TE absorption spectra of HNA and AE-HNA structures capsulated in a 350-nm-thick SiO_2_ layer, respectively. In comparison with HNA, the AE-HNA exhibits significantly higher absorption over nearly the whole wavelength range due to multiple reflection and re-absorption over a wide spectrum. However, the absorption enhancement at different wavelengths is not equal, which is probably due to the film interference. At some wavelengths, the optical path difference satisfies constructive interference. Hence, the reflection is enhanced and the absorption is suppressed, corresponding to the absorption valley. For some wavelengths, the optical path difference satisfies constructive interference and destructive interference. Hence, the reflection is suppressed and the absorption is enhanced, corresponding to the absorption maximum. Figure 6c shows the cross-section optical generation profiles under AM 1.5 G illumination in the HNA and AE-HNA structures, respectively. Apparently, the NW in the AE-HNA structure absorbs more photons in comparison with the HNA structure. In the HNA structure, strong absorption concentrates in the NW center, which is mainly from the light directly impinging on the NW body, while in the AE-HNA structure, the strong absorption region covers the whole top half of the NW cross-section and a large part of the bottom half. It is indicated by comparison that the multiple reflection and re-absorption in the AE-HNA structure significantly enhance the absorption in the top half. For the bottom part near the MgF_2_ substrate, the re-absorption is weak as the reflected light from the SiO_2_/MgF_2_ interface is difficult to reach the NW bottom. In future studies, the material and shape of the substrate and encapsulant need to be optimized to enhance the re-absorption by the NW bottom [30].

**Figure 7:**
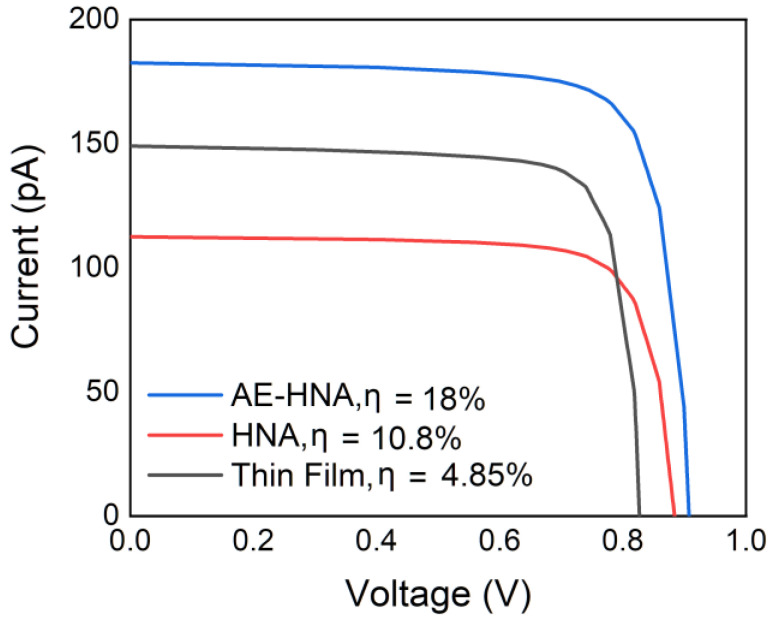
The current–voltage characteristics of the AE-HNA, HNA, and thin-film structures.

Figure 7 shows the current–voltage characteristics of the AE-HNA, HNA, and thin-film structures. The AE-HNA structure yields an I_sc_ of 183 pA and a V_oc_ of 0.91 V, resulting in a high efficiency of 18%, 1.7 times higher than that of the HNA structure and 3.7 times higher than that of the thin-film counterpart. Even considering the void space, the AE-HNA structure still yields an efficiency of 7.2%, 1.5 times higher than that of the thin-film counterpart. It should be pointed out that with the same thickness, the volume of the NW array is only 31.4% that of the thin-film structure, demonstrating the potential of the AE-HNA structure in ultra-thin high-performance NW solar cells with very low material cost.

## 4. Conclusions

In summary, a horizontally aligned GaAs p–i–n NW array solar cell is proposed and studied. The HNA yields a remarkable efficiency of 10.8% with a radius of 90 nm and period of 5 radius, more than twice that of its thin-film counterpart with the same equivalent thickness. To further enhance the absorption, the ultra-thin array is placed on a low-refractive-index MgF_2_ substrate and capsulated in SiO_2_, which enables multiple reflection and reabsorption of light due to the refractive index difference between air/SiO_2_ and SiO_2_/MgF_2_. The absorption-enhancement structure increases the absorption over a broad wavelength range, resulting in a maximum conversion efficiency of 18%. This work provides an effective way to achieve ultra-thin high-efficiency solar cells with very low III–V material cost.

## Figures and Tables

**Table 1:** Key simulation parameters.

Parameters	Electron (Hole)
Minimum mobility	2.136 × 10^3^ (21.48) cm^2^/Vs
SRH lifetime	1 ns
Radiative recombination coefficient	7.2 × 10^−^^10^ cm^3^/s
Auger recombination coefficient	1.9 × 10^−31^ (1.2 × 10^−31^) cm^6^/s

## References

[1] Hochbaum A.I., Yang P. (2010). Semiconductor Nanowires for Energy Conversion. Chem. Rev..

[2] Garnett E.C., Brongersma M.L., Cui Y., McGehee M.D. (2011). Nanowire Solar Cells. Annu. Rev. Mater. Res..

[3] Borgstrom M.T., Wallentin J., Heurlin M., Fält S., Wickert P., Leene J., Magnusson M.H., Deppert K., Samuelson L. (2010). Nanowires with Promise for Photovoltaics. IEEE J. Sel. Top. Quantum Electron..

[4] Otnes G., Borgström M.T. (2017). Towards high efficiency nanowire solar cells. Nano Today.

[5] Åberg I., Vescovi G., Asoli D., Naseem U., Gilboy J.P., Sundvall C., Dahlgren A., Svensson K.E., Anttu N., Björk M.T. (2015). A GaAs Nanowire Array Solar Cell With 15.3% Efficiency at 1 Sun. IEEE J. Photovolt..

[6] Wu Y., Yan X., Zhang X., Ren X. (2018). Photovoltaic Performance of a Nanowire/Quantum Dot Hybrid Nanostructure Array Solar Cell. Nanoscale Res. Lett..

[7] Luo Y., Yan X., Zhang J., Li B., Wu Y., Lu Q., Jin C., Zhang X., Ren X. (2018). A graphene/single GaAs nanowire Schottky junction photovoltaic device. Nanoscale.

[8] Tian B., Kempa T.J., Lieber C.M. (2009). Single nanowire photovoltaics. Chem. Soc. Rev..

[9] Krogstrup P., Jørgensen H.I., Heiss M., Demichel O., Holm J.V., Aagesen M., Nygård J., Fontcuberta i Morral A. (2013). Single-nanowire solar cells beyond the Shockley–Queisser limit. Nat. Photonics.

[10] Han N., Yang Z.-X., Wang F., Dong G., Yip S., Liang X., Hung T.F., Chen Y., Ho J.C. (2015). High-Performance GaAs Nanowire Solar Cells for Flexible and Transparent Photovoltaics. ACS Appl. Mater. Interfaces.

[11] Tian B., Zheng X., Kempa T.J., Fang Y., Yu N., Yu G., Huang J., Lieber C.M. (2007). Coaxial silicon nanowires as solar cells and nanoelectronic power sources. Nature.

[12] Kelzenberg M.D., Turner-Evans D., Kayes B.M., Filler M.A., Putnam M.C., Lewis N.S., Atwater H.A. (2008). Photovoltaic Measurements in Single-Nanowire Silicon Solar Cells. Nano Lett..

[13] Colombo C., Heiβ M., Grätzel M., Morral A.F.I. (2009). Gallium arsenide p-i-n radial structures for photovoltaic applications. Appl. Phys. Lett..

[14] Han N., Wang F., Yip S., Hou J.J., Xiu F., Shi X., Hui A.T., Hung T., Ho J.C. (2012). GaAs nanowire Schottky barrier photovoltaics utilizing Au–Ga alloy catalytic tips. Appl. Phys. Lett..

[15] Gutsche C., Lysov A., Braam D., Regolin I., Keller G., Li Z.-A., Geller M., Spasova M., Prost W., Tegude F.-J. (2011). n-GaAs/InGaP/p-GaAs Core-Multishell Nanowire Diodes for Efficient Light-to-Current Conversion. Adv. Funct. Mater..

[16] Ye Y., Dai Y., Dai L., Shi Z., Liu N., Wang F., Fu L., Peng R., Wen X., Chen Z. (2010). High-Performance Single CdS Nanowire (Nanobelt) Schottky Junction Solar Cells with Au/Graphene Schottky Electrodes. ACS Appl. Mater. Interfaces.

[17] Tang J., Huo Z., Brittman S., Gao H., Yang P. (2011). Solution-processed core–shell nanowires for efficient photovoltaic cells. Nat. Nanotechnol..

[18] Zhong Z., Li Z., Gao Q., Li Z., Peng K., Li L., Mokkapati S., Vora K., Wu J., Zhang G. (2016). Efficiency enhancement of axial junction InP single nanowire solar cells by dielectric coating. Nano Energy.

[19] Song K.-D., Kempa T.J., Park H.-G., Kim S.K. (2014). Laterally assembled nanowires for ultrathin broadband solar absorbers. Opt. Express.

[20] Kim S.K., Day R., Cahoon J.F., Kempa T.J., Song K.-D., Park H.-G., Lieber C.M. (2012). Tuning Light Absorption in Core/Shell Silicon Nanowire Photovoltaic Devices through Morphological Design. Nano Lett..

[21] Nowzari A., Heurlin M., Jain V., Storm K., Hosseinnia A., Anttu N., Borgström M.T., Pettersson H., Samuelson L. (2015). A Comparative Study of Absorption in Vertically and Laterally Oriented InP Core–Shell Nanowire Photovoltaic Devices. Nano Lett..

[22] Hosseinnia A., Anttu N. (2015). Absorption through a coupled optical resonance in a horizontal InP nanowire array. Photonics Res..

[23] Levinshtein M., Rumyantsev S., Shur M. (1999). Handbook Series on Semiconductor Parameters, Ternary, and Quaternary III–V Compounds.

[24] Malitson I.H. (1965). Interspecimen Comparison of the Refractive Index of Fused Silica. J. Opt. Soc. Am..

[25] Dodge M.J. (1984). Refractive properties of magnesium fluoride. Appl. Opt..

[26] Arora N., Hauser J., Roulston D. (1982). Electron and hole mobilities in silicon as a function of concentration and temperature. IEEE Trans. Electron Dev..

[27] Wu Y., Yan X., Zhang X., Ren X. (2015). Enhanced photovoltaic performance of an inclined nanowire array solar cell. Opt. Express.

[28] Svensson J., Anttu N., Vainorius N., Borg M., Wernersson L.-E. (2013). Diameter-Dependent Photocurrent in InAsSb Nanowire Infrared Photodetectors. Nano Lett..

[29] Cao L., Fan P., Vasudev A.P., White J.S., Yu Z., Cai W., Schuller J.A., Fan S., Brongersma M.L. (2010). Semiconductor Nanowire Optical Antenna Solar Absorbers. Nano Lett..

[30] Yan X., Liu H., Sibirev N., Zhang X., Ren X. (2020). Performance Enhancement of Ultra-Thin Nanowire Array Solar Cells by Bottom Reflectivity Engineering. Nanomaterials.

